# Evaluation of Early Retinal Nerve Injury in Type 2 Diabetes Patients Without Diabetic Retinopathy

**DOI:** 10.3389/fendo.2020.475672

**Published:** 2020-09-29

**Authors:** Xiuhua Jia, Zhijian Zhong, Tiancheng Bao, Shasha Wang, Ting Jiang, Yanling Zhang, Qigen Li, Xiang Zhu

**Affiliations:** ^1^Department of Ophthalmology, Third Affiliated Hospital of Sun Yat-sen University, Guangzhou, China; ^2^Department of Radiology, Third Affiliated Hospital of Sun Yat-sen University, Guangzhou, China; ^3^Department of Ultrasonography, Third Affiliated Hospital of Sun Yat-sen University, Guangzhou, China; ^4^Department of Infectious Diseases, Third Affiliated Hospital of Sun Yat-sen University, Guangzhou, China

**Keywords:** diabetic retinopathy, optical coherence tomography, ganglion cells (GC), nerve fiber layer, glycosylated hemoglobin (HbA1c), plasma glucose

## Abstract

**Objectives:** To investigate the damage to the retinal nerve fiber layer (RNFL) and ganglion cell complex layer (GCL+) in diabetic patients without retinal microangioma and to determine the kind of nerve damage more likely to indicate early injury.

**Subjects and Methods:** We included 360 patients (360 eyes) with type 2 diabetes mellitus and 168 healthy volunteers (168 eyes). Patients with retinal microangioma were excluded by fundus fluorescein angiography (FFA). The parameters around the optic disc and macular area were measured by optical coherence tomography (OCT).

**Results:** The peripapillary RNFL thickness was thinner in the temporal (72.98 ± 13.76 μm, *P* < 0.0001) and inferior (120.71 ± 21.43 μm, *P* = 0.0103) sectors in patients with no diabetic retinopathy (NDR) compared to healthy controls. The reduction of retinal thickness in the macular region was prominent in the inferior sector in patients (34.74 ± 4.92 μm, *P* < 0.0001) compared to normal controls. Thinning of GCL+ in the second region of the macular area was significant in patients with NDR compared to normal controls (*P* < 0.05). However, no difference in the GCL+ and retinal thicknesses of the central macular region was observed between the patients with NDR and healthy controls. Using the 5th percentile (P5) of normal controls as the reference value, we found that the parameters with the highest indices in patients with NDR were the inferior and temporal peripapillary RNFL thickness (13.0%), the inferior RNFL thickness in the macular area (20%), the inferior retinal thickness in the outer ring of the macular area (10.8%), and the inferior GCL+ thickness in the macular area (10.6%). The GCL+ and RNFL thicknesses in the central macular area accounted for the smallest proportion in P5 of normal controls (3%).

**Conclusions:** Retinal nerve injury can occur in patients without retinal microangioma. The inferior RNFL in the macular area and the inferior and temporal peripapillary RNFL were most sensitive to glucose damage. These areas might be associated with early detection of diabetic retinopathy (DR) as they are more likely to indicate early damage.

## Introduction

Diabetic retinopathy (DR) is the leading cause of blindness in working adults. There are ~190 million people with DR worldwide (1). The overall prevalence rate of DR among adults with diabetes mellitus (DM) is 34.6% (2). Microvascular disease and its neurological complications are known to be serious complications associated with DR. DR is thought to be caused by microvascular disorders. It is reported that duration of diabetes, blood pressure, and glycosylated hemoglobin are the major risk factors of DR (3). It is not clear whether neurodegeneration is an independent factor or a consequence of the damage to the retinal vascular system. Whether neural or vascular defects occur first during the early stage of DR is undetermined. However, accumulating evidence suggests that neurodegeneration of the retina occurs before the occurrence of clinically detectable microvascular damage (4). Neurodegeneration is an early component of DR. Recent studies have shown that the thickness of the retinal nerve fiber layer (RNFL) can decrease before the onset of DR, which can be detected by ophthalmoscopy. However, changes in the RNFL and ganglion cell layer (GCL), which are more likely to indicate the degree of disease damage, have not been previously reported. Thus, using cross-sectional analysis, the purpose of the present study is to observe whether retinal neurodegeneration preceded the first signs of microvascular lesions (i.e., microangioma) detected via fundus fluorescein angiography (FFA) in diabetic patients as well as to observe the characteristics of neurodegenerative changes. In addition, specific alterations to the RNFL and ganglion cells in the macular and peripapillary sectors were also explored. Our investigation is of high significance for the evaluation of early diabetic retinal nerve injury.

## Subjects and Methods

### Participants

In the present cross-sectional observational study, 360 patients with type 2 DM (T2DM) and 168 healthy control subjects were recruited from the Third Affiliated Hospital of Sun Yat-Sen University between January 2016 and January 2018. Patients with T2DM were diagnosed in the Department of Endocrinology in the same hospital. The study was approved by the Ethics Committee of the Third Affiliated Hospital of Sun Yat-Sen University (Guangzhou, China, [2015]02-423-01) and was conducted according to the Declaration of Helsinki. Written informed consent was obtained from all participants. The inclusion criteria of patients in our study are as follows: Patients with type 2 DM and have a disease course of 5–30 years; Patients with poor blood glucose control (glycosylated hemoglobin ≥6.5%); Male or female. Age between 18 and 65; Patients without microangioma. One eye from each participant was included in the study. A single eye was randomly selected if both eyes were eligible for study inclusion. According to the International Diabetic Retinopathy Severity Scale in 2002, patients without microangioma were initially screened using direct or indirect ophthalmoscopy after pupil dilation and then included in the group of patients undergoing FFA examination. Patients without microangioma changes undergoing FFA examination were included in the present study. All of the participants enrolled in the present study underwent a systematic ophthalmic examination. The exclusion criteria for all subjects were any other ocular disease that might affect retinal nerve injury (e.g., glaucoma and refractive error exceeding 3 diopters), optic neuropathy, age-related macular degeneration, retinal and choroidal disease, and retinal artery/vein occlusion. In addition, we excluded individuals with hypertension, hematopathy, neuropathy, and other systemic diseases causing retinal nerve changes. All participants were examined for best corrected visual acuity. Patients with keratopathy, cataract, and vitreous hemorrhage were also excluded.

### Methods

All participants underwent routine ophthalmic examination, including slit lamp, intraocular pressure, and fundus examinations using direct or indirect ophthalmoscopy after pupil dilation and optical coherence tomography (OCT) examination. Patients with DM also underwent FFA examination(Zeiss VISUCAM524, Oberkochen, Germany).

#### OCT Image Acquisition and Analysis (TOPCON3D-OCT2000, Tokyo, Japan)

Image acquisition was performed through the following four scanning modes:

##### The 3D disc mode

**Figure 1:**
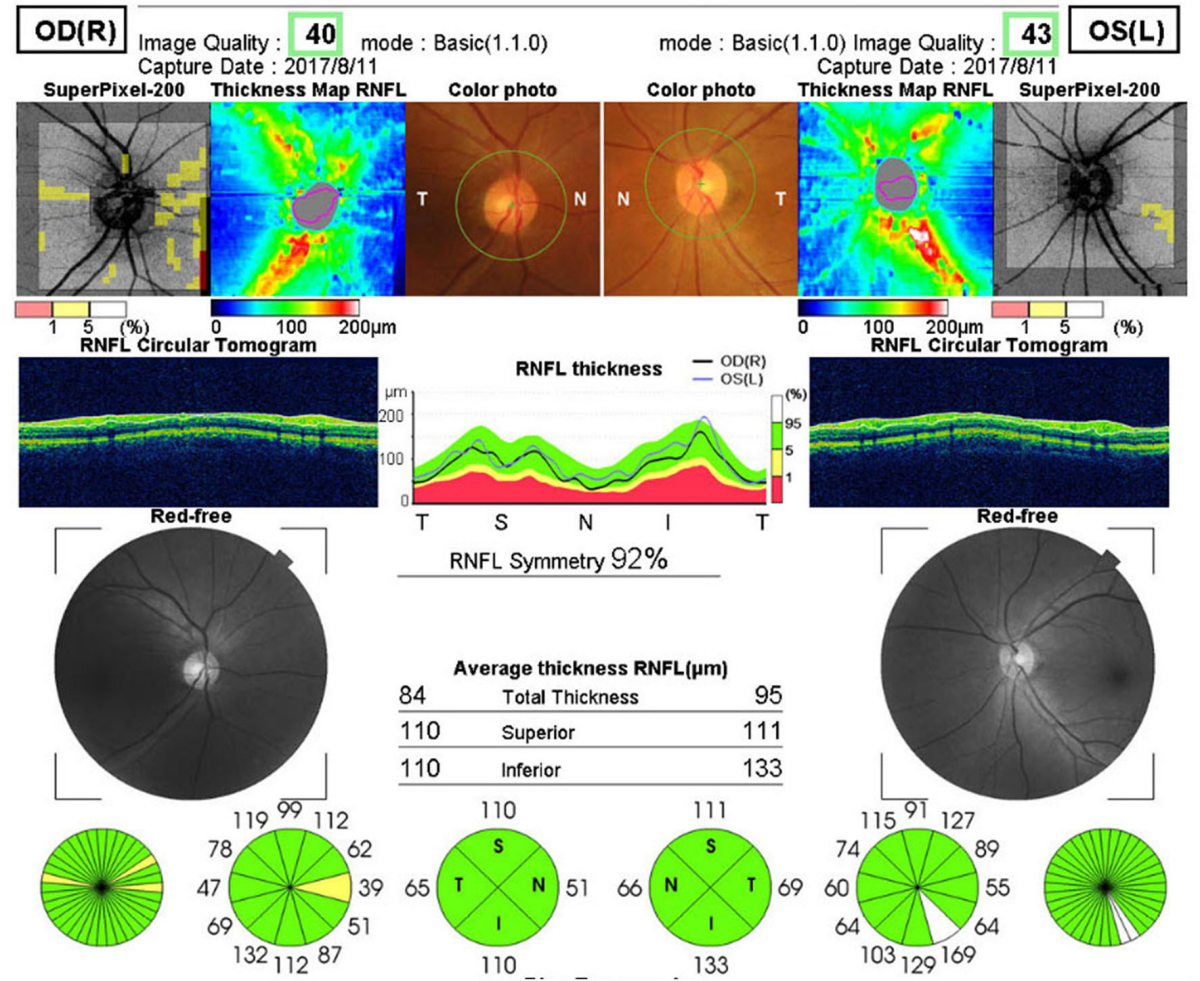
3D Disc mode. The thickness of the nerve fiber layers in all four peripapillary sectors (6 × 6-mm area around the optic disc).

(6 × 6-mm area around the optic disc, Figure 1) was used to scan the thickness of the nerve fiber layers in all four peripapillary sectors.

##### The 3D wide mode

**Figure 2:**
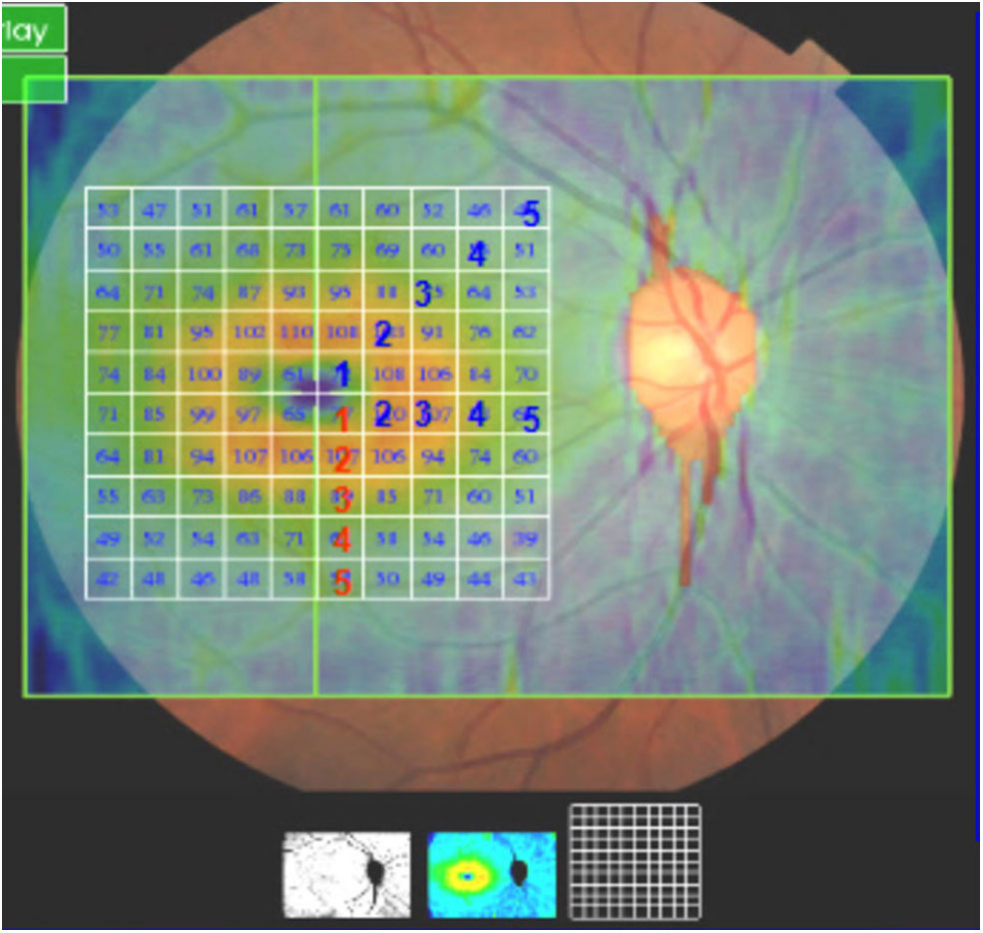
3D Wide mode. The thickness of the ganglion cell complex layer (GCL+), which include the ganglion cell layer and inner plexus layer in the macular area (mGCL and mIPL), including the central region, the second region, and the third region (12 × 9-mm in the macular area).

(12 × 9-mm in the macular area, Figure 2) was used to scan the thickness of the ganglion cell complex layer (GCL+), which include the ganglion cell layer and inner plexus layer in the macular area (mGCL and mIPL), including the central region, the second region, and the third region. Statistical analysis was performed according to the parameters of the tempo superior, nasal superior, nasal inferior, and tempo inferior retinal quadrants.

##### The 3D macula mode

**Figure 3:**
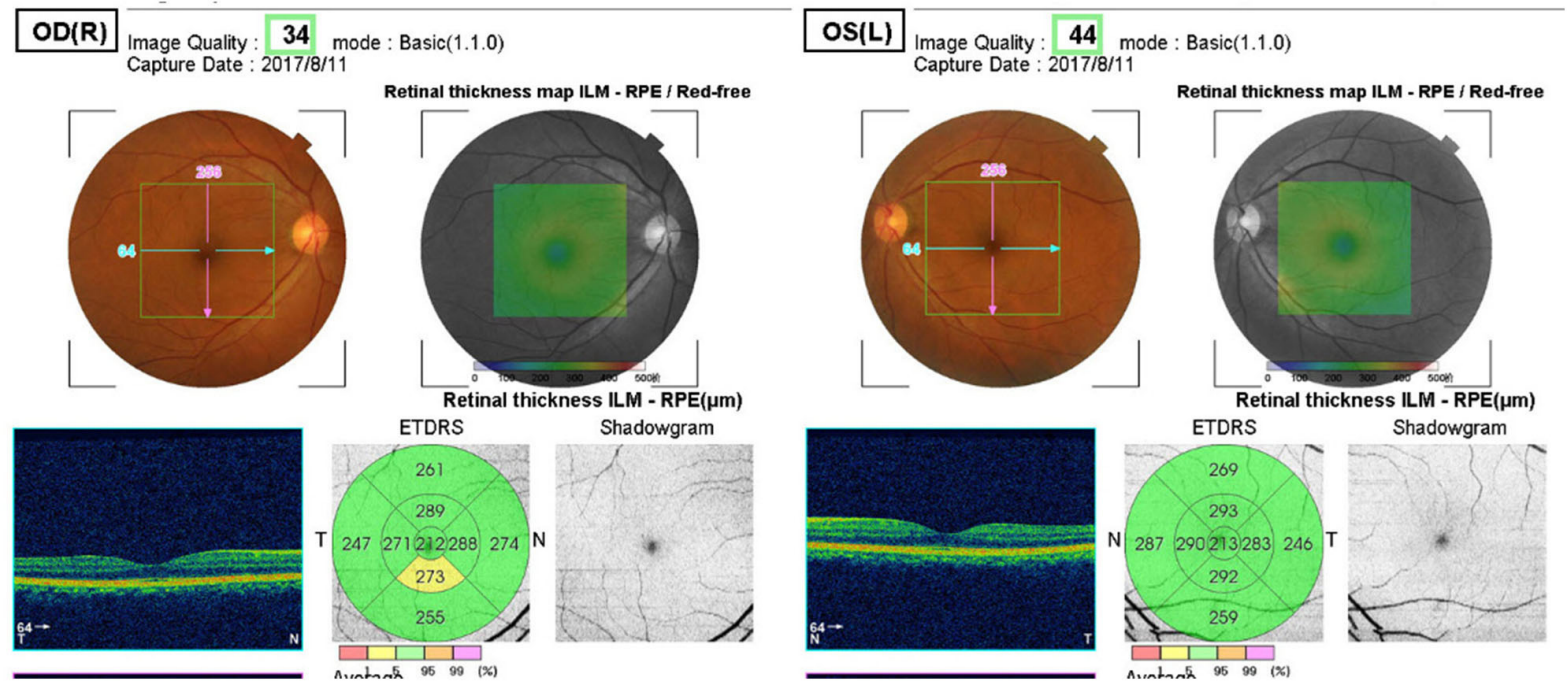
3D Macula mode. The retinal thickness of the macular area, including the central area, the inner ring, and the outer ring of the macular area (6 × 6-mm in the macular area).

(6 × 6-mm in the macular area, Figure 3) was used to scan the retinal thickness of the macular area, including the central area, the inner ring, and the outer ring of the macular area. Statistical analysis was performed according to the parameters of the superior (S), inferior (I), nasal (N), and temporal (T) quadrants.

##### The 3D macula (v) mode

**Figure 4:**
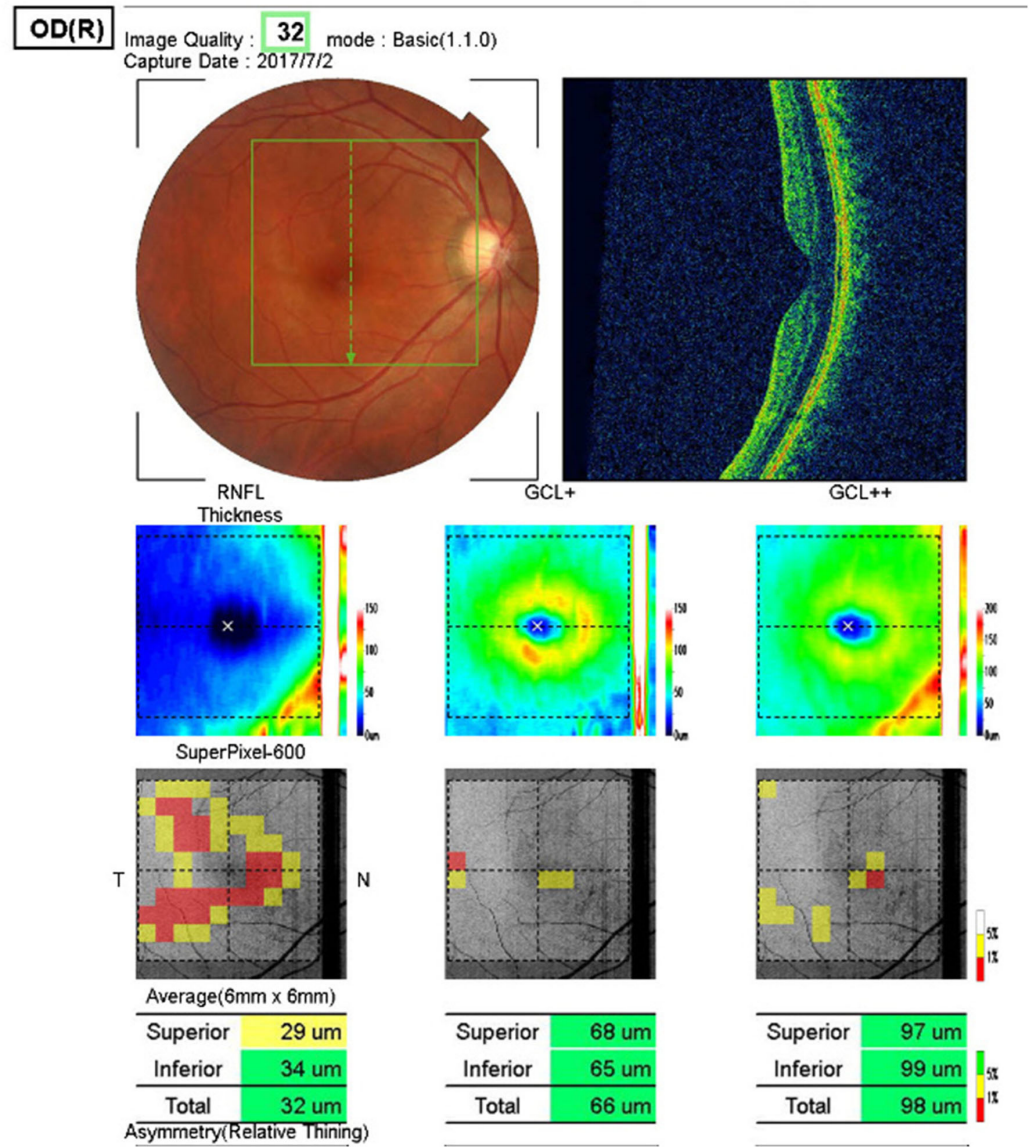
3D Macula (v) mode. The GCL+ layer and the nerve fiber layer in the macular area. (7 × 7-mm in the macular area).

(7 × 7-mm in the macular area, Figure 4) was used to scan the GCL+ layer and the nerve fiber layer in the macular area.

### Statistical Analysis

All statistical analyses were performed using the SPSS 22.0 software (Chicago, IL, USA), and all data were expressed as mean ± standard deviation within the 5^th^ percentile (P5). Statistical differences between the control and patient groups were analyzed using one-way analysis of variance. *P* < 0.05 was considered statistically significant.

## Results

### Subjects

In the present study, we included 360 eyes from 360 patients with T2DM (214 men and 146 women) and 168 eyes from 168 healthy controls (102 men and 66 women). The patients have a disease course of 5–30 years (average, 10.7 ± 6.5 years). The mean ages in the T2DM and control groups were 55.43 ± 8.155 and 54.16 ± 8.743 years, respectively (Table 1). No statistical differences were observed between the gender (*P* = 0.477) and ages (*P* = 0.282) of patients and healthy individuals. All of the patients that we screened had normal intraocular pressure and visual acuity (0.89 ± 0.14) (Table 1). However, the diabetic patients showed poor blood glucose control (glycosylated hemoglobin, 7.04 ± 1.5%) (Table 1). The characteristics of the OCT results are shown in Table 2.

**Table 1:** Characteristics of the patients with T2DM.

**Patients with T2DM *n* = 360**	**Age(year)**	**Vision**	**GHbA1c(%)**
	55.43 ± 8.155	0.89 ± 0.14	7.04 ± 1.5

*T2DM, type 2 diabetes mellitus; GHbA1c, Glycosylated Hemoglobin A1c*.

**Table 2:** Characteristics for the OCT studies in the diabetic and control groups.

**Statistical objects**			**Control**		**T2DM with NDR**		***P*-value**
			**Mean**	**Standard deviation**	**Mean**	**Standard deviation**	
GCL+ thickness in the macular region (3D Wide mode)	Center (μm)		55.85	6.96	54.44	7.09	0.5488
	Second region	Tempo superior (μm)	90.49	6.58	88.99	8.04	0.4710
		Nasal superior (μm)	97.23	6.59	90.19	9.48	0.0019
		Nasal inferior (μm)	92.02	6.50	89.59	8.29	0.0274
		Tempo inferior (μm)	91.63	6.48	90.21	8.35	0.5444
	Third region	Tempo superior (μm)	81.24	5.93	80.65	7.05	0.9972
		Nasal superior (μm)	87.27	6.80	85.71	8.33	0.3981
		Nasal Inferior (μm)	84.33	6.65	83.09	8.84	0.7197
		Tempo inferior (μm)	81.4	5.80	80.56	7.97	0.9617
Peripapillary RNFL thickness	Superior (μm)		119.3	14.77	116.4	18.54	0.3429
	Nasal (μm)		74.33	10.92	73.3	12.76	0.9956
	Inferior (μm)		126.1	12.97	120.7	21.43	0.0103
	Temporal (μm)		82.19	15.75	72.98	13.76	<0.0001
Retinal thickness in the macular region	Center (μm)		228	17.74	230	19.85	0.9631
	Inner ring	Superior (μm)	300.3	13.80	300.1	15.57	>0.9999
		Nasal (μm)	291	16.19	289.8	18.20	0.9990
		Inferior (μm)	285.7	16.38	286	18.21	>0.9999
		Temporal (μm)	293.4	15.95	293.4	17.27	>0.9999
	Outer ring	Superior (μm)	262.2	13.42	262.8	14.57	>0.9999
		Nasal (μm)	268.1	20.41	268.1	20.70	>0.9999
		Inferior (μm)	263.5	13.52	262.8	16.46	>0.9999
		Temporal (μm)	267.9	20.47	265.9	20.65	0.9519
RNFL thickness in the macular region	Superior (μm)		33.45	3.64	32.97	3.95	0.6560
	Inferior (μm)		36.91	4.27	34.74	4.92	<0.0001
	Total (μm)		34.89	3.72	33.95	4.06	0.1310
GCL+ thickness in the macular region	Superior (μm)		70.97	4.61	70.76	5.14	0.9705
[The 3D Macula(v) mode]	Inferior (μm)		69.09	4.39	68.19	5.31	0.2695
	Total (μm)		70.07	4.48	69.53	5.06	0.6902

*NDR, no diabetic retinopathy; RNFL, retinal nerve fiber layer; T2DM, type 2 diabetes mellitus; GCL+, thickness of the ganglion cell complex layer, including mIPL and mGCL*.

According to the Early Treatment of Diabetic Retinopathy Study, the macular region is divided into three concentric circles, central (≤ 1 mm in diameter), inner ring (>1–3 mm), and outer ring (Figure 3); The central area, second area, and third area of the macula are shown in Figure 2. (The area of each grid area is 0.6 * 0.6 mm = 0.36 mm^2^).

### Peripapillary RNFL Thickness

**Figure 5:**
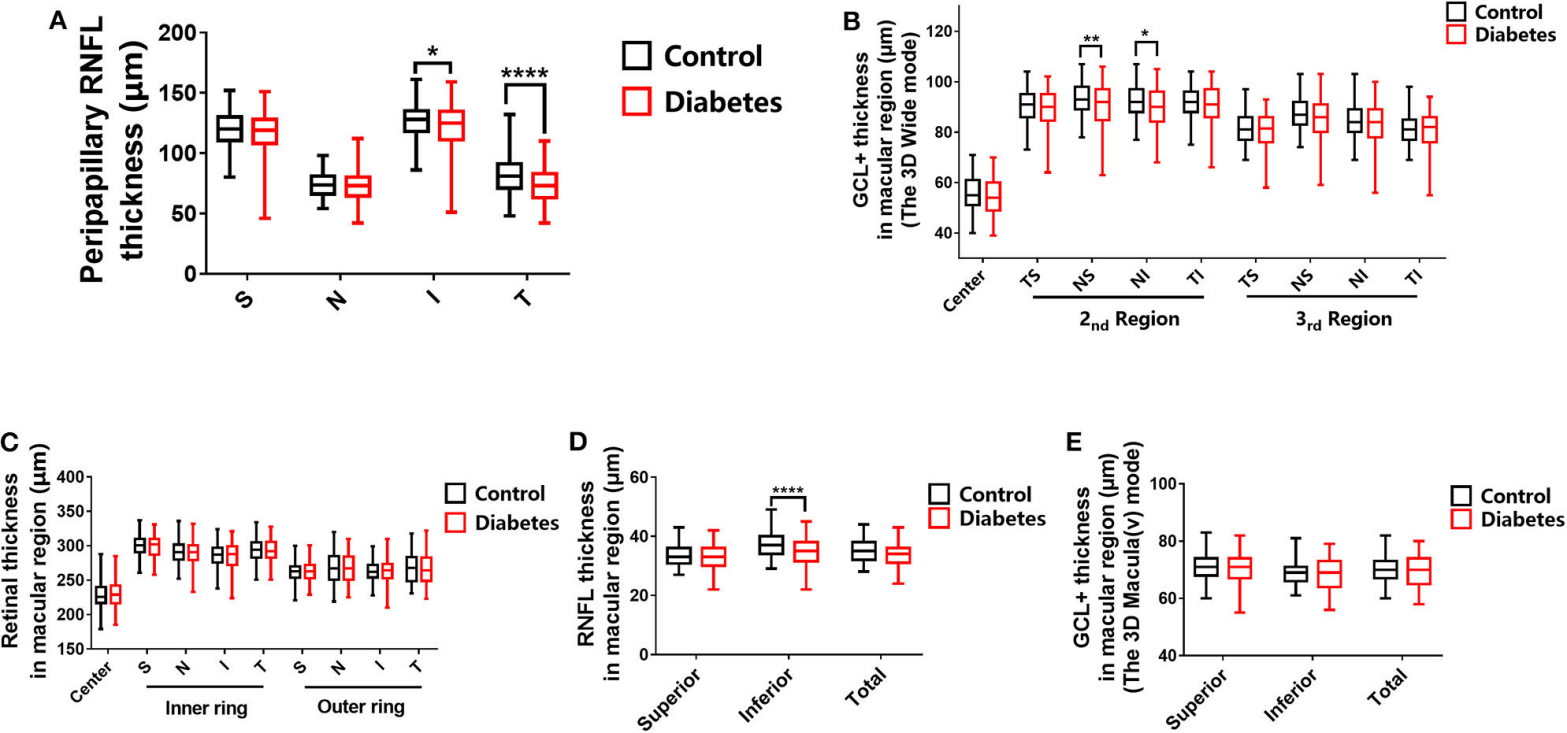
Statistical analyses of all the scan modes. **(A)** Peripapillary RNFL thickness. **(B)** Ganglion cell complex layer (GCL+) thickness in the macular region in the 3D wide mode. **(C)** Retinal thickness in the macular region. **(D)** RNFL thickness in the macular region. **(E)** GCL+ thickness in the macular region in the 3D Macula (v) mode. The stars represent the range of the *P* value. *P (0.05, 0.01), **P (0.01, 0.005), and ****P (< 0.001).

In the present study, we showed that the peripapillary RNFL thickness was significantly reduced in patients with preclinical DR compared to healthy controls in the inferior quadrant (*P* = 0.0103) and the temporal quadrant (*P* < 0.0001). However, there were no significant differences between the RNFL thicknesses of healthy controls and T2DM patients with no DR (NDR) in the superior quadrant (*P* = 0.3429) and the nasal quadrant (*P* = 0.9956; Table 2 and Figure 5A).

### GCL+ in the Macular Area Obtained Using the 3D Wide Mode

In the present study, we show that the GCL+ in the nasal superior (NS) quadrant and the nasal inferior (NI) quadrant of the second macular area was significantly decreased in T2DM patients with NDR compared to healthy controls (*P* = 0.0019 and *P* = 0.0274, respectively). However, differences were not observed between the other regions of healthy controls and T2DM patients with NDR (Table 2 and Figure 5B).

### Retinal Thickness in All Macular Regions

Ophthalmic examination showed that that there were no significant differences (*P* > 0.05) between all macular regions of healthy controls and T2DM patients with NDR (Table 2 and Figure 5C).

### RNFL Thickness in the Macular Area Obtained Using the 3D Macula (v) Mode

The RNFL thickness in the inferior (I) quadrant of the macular area was 34.74 ± 4.92 μm in the T2DM group and 36.91 ± 4.27 μm in the control group. The RNFL thickness in the I quadrant of the macular region was significantly lower in T2DM patients with NDR compared to healthy controls (*P* < 0.0001; Table 2 and Figure 5D).

### GCL+ Thickness in the Macular Region Obtained Using the 3D Macula (v) Mode

We did not observe any significant differences between the GCL+ thickness of any macular region of healthy controls and T2DM patients with NDR (Table 2 and Figure 5E).

### Statistical Results of T2DM Patients With NDR in P5 of Normal Controls

Using the P5 of healthy controls as a reference value, the indices having a high proportion of T2DM patients with NDR within the reference range are the inferior (I) and temporal (T) peripapillary RNFL thickness (13.0%), the I retinal thickness in the outer ring of the macular area (10.8%), the inferior RNFL thickness in the macular area (20%), and the I GCL+ thickness in the macular area (10.6%). The GCL+ thickness and RNFL thickness of T2DM patients with NDR in the central macular area accounted for the smallest proportion of patients with thickness within the reference range (3%; Table 3).

**Table 3:** Statistical object percentage of T2DM with NDR in P5 of normal control.

**Statistical objects in the control groups**			**Mean**	**Median**	**P5**	**Percentage of T2DM with NDR in P5 of normal controls %**
GCL+ thickness in the macular	Center (μm)		55.85	55	43	3.0
Region (3D Wide mode)	Second region	Tempo inferior (μm)	90.49	91	78	9.0
		Nasal superior (μm)	93.23	93	81	13.2
		Nasal inferior (μm)	92.02	92	79	9.6
		Tempo inferior (μm)	91.63	92	78	11.4
	Third region	Tempo inferior (μm)	81.24	81	71	6.6
		Nasal superior (μm)	87.27	87	77	10.8
		Nasal inferior (μm)	84.33	84	74	15
		Tempo inferior (μm)	81.4	81	73	13.8
Peripapillary	Superior (μm)		119.3	120	92	8.0
RNFL thickness	Nasal (μm)		74.33	73.5	47	3.1
	Inferior (μm)		126.1	128	101	13.0
	Temporal (μm)		82.19	81	59	13.0
Retinal thickness in the macular region	Center (μm)		228	226	199	3.0
	Inner ring	Superior (μm)	300.3	302	277	7.8
		Nasal (μm)	291	291	262	6.6
		Inferior (μm)	285.7	287	254	3.6
		Temporal (μm)	293.4	294	267	8.4
	Outer ring	Superior (μm)	262.6	263	241	7.8
		Nasal (μm)	268.1	267	239	7.8
		Inferior (μm)	263.5	262	243	10.8
		Temporal (μm)	267.9	268	238	7.2
RNFL thickness in the macular region	Superior (μm)		33.45	33	28	5.6
	Inferior (μm)		36.91	37	31	20
	Total (μm)		34.89	35	29	9.4
GCL+ thickness in the macular region (3D Macula (v) mode)	Superior (μm)		70.97	71	63	6.3
	Inferior (μm)		69.09	69	62	10.6
	Total (μm)		70.07	70	63	9.4

*NDR, no diabetic retinopathy; RNFL, retinal nerve fiber layer; T2DM, type 2 diabetes mellitus; GCL+, thickness of the ganglion cell complex layer, including mIPL and mGCL*.

According to the Early Treatment of Diabetic Retinopathy Study, the macular region is divided into three concentric circles, central (≤ 1 mm in diameter), inner ring (>1–3 mm), and outer ring (Figure 3); The central area, second area, and third area of the macula are shown in Figure 2. (The area of each grid area is 0.6 ^*^ 0.6 mm = 0.36 mm^2^).

## Discussion

With the aging of the population and the increasing prevalence of diabetes, the prevalence of DR has also been increasing (5). It is estimated that by 2050, the number of people with diabetes in the United States will reach 16 million (6). DR is a serious complication of diabetes, which may lead to irreversible vision loss owing to advanced severe proliferative retinopathy and intractable macular edema, and it is one of the most devastating acquired vascular complications affecting the life quality of diabetic patients. Understanding the pathophysiology of DR is not only conducive to disease management but also crucial for the development of new therapeutic strategies.

DR involves changes to blood vessels and nerve tissue. Retinal degeneration has been widely confirmed in animal models and clinical studies. The loss of the nerve fiber layer and inner plexus layer tissue is the most common clinical manifestation (7). An increasing number of studies have analyzed the RNFL through non-invasive research methods such as OCT. Recently, the clinical diagnosis of DR mainly depends on the detection of vascular lesions by fundus examination. It was found that loss of the inner retinal layer can occur in patients without vascular lesions and macular edema upon examination by OCT. Currently, FFA is the golden standard for the diagnosis of DR (8). In the present study, microangioma was diagnosed via FFA examination. We found that retinal neurodegeneration was an early component of DR, which precedes microangioma. Evidence of retinal nerve injury in patients without DR suggested that degenerative loss of the inner retinal nerve tissue might occur relatively early in the period preceding the occurrence of DR (9, 10). Recently, it has been reported that the thinning of RNFL, GCL+, and internal plexus layer occurs progressively at a rate of 0.29 μm/year, preceding the occurrence of clinically detectable vascular lesions (11). Research focusing on the neurodegenerative changes associated with DR is beneficial for the identification of new therapeutic targets and neuroprotectants, which are expected to prevent the occurrence of DR.

OCT is an important tool in the early detection of DR damage. We assessed the neurologic changes around the optic disc and macular area with the four scanning methods mentioned in the Methods section above. Before the occurrence of DR in diabetic patients, the thickness of GCL+ and retinal thickness in the center of the macular area were identified as the least vulnerable indices. Irrespective of the region, the I quadrant of each index was identified to be more sensitive to blood glucose fluctuations. However, among all indices, the inferior RNFL thickness in the macular area and the inferior and temporal peripapillary RNFL thicknesses were identified as the most sensitive indicators. These parameters are associated with early injury and might be important biomarkers for the early detection of DR.

We believe that the mentioned characteristics of nerve injury are closely related to the distribution of nerve fiber layers in the retina. The RNFL thickness of each region is different. In healthy individuals, RNFL was thickest in the temporal side of the superior and inferior quadrants (12). In particular, RNFL was denser in the arcuate area of the temporal side (13). In the present study, RNFL was damaged at an early stage in these regions. However, RNFL in the nasal quadrant was sparse and showed later signs of damage. This could indicate a direct correlation between RNFL density and sensitivity to blood glucose fluctuations and vulnerability to injury. These changes in the anatomical layers of nerve fibers might provide new insights into the nerve damage associated with DR.

There is a special nerve and blood vessel homeostasis in the retina. It has been shown in previous studies that the basement membrane of capillaries in the DR area is significantly thickened, which leads to a decrease of oxygen diffusion from capillaries to tissues, because the tissue adjacent to retinal arterioles is usually supplied with oxygen by the arterioles in the non-capillary area through diffusion. Retinal changes that occur as a result of diabetes are the shrinkage of the tissue area and reduction of oxygen consumption, resulting in increased retinal arterial oxygen saturation (14). It was found that the blood oxygen saturation of the temporal side of the retina in early diabetic patients was higher than that in the healthy control group (15, 16). In addition, the characteristics of retinal nerve injury identified in the present study are consistent with these findings from previous studies, which offers a partial explanation for the concept of “nerve-vascular unit.” The relationship between vascular pathological changes and neurodegeneration in DR remains controversial. The causal relationship or independence between vascular pathological changes and retinal neurodegeneration in DR remains to be established (17, 18). The imbalance of nerve and blood vessel homeostasis results in important pathophysiological changes in retinal neurovascular diseases and initiates the occurrence of DR.

GCL+ represents the ganglion cell layer and inner plexus layer, where the retinal ganglion cells and dendrites are located. On the other hand, RNFL represents the axons. Our findings showed that, although there was no difference in GCL+ in the macular area during early stages, there was a significant difference in the nerve fiber layer. To some extent, this indicates that the nerve fiber layer (axons) is damaged initially. Diabetes is associated with peripheral nerve disease. The most common phenomenon is the slow progression of distal axonal neuropathy. DM causes irreversible damage to the nerves through degeneration of neurons and axons. In the present study, we found that the early abnormality observed in DR is the axonal degeneration of nerve fibers. A particularly common phenomenon in T2DM is changes to the structure and gene expression of maternal neurons after peripheral axon injury, also known as retrograde response. Regulatory damage can activate changes in neurons and lead to impaired delayed functional recovery (19). Finally, the loss of neurons occurs within a few weeks after injury. In the present study, RNFL was damaged first, which is inconsistent with the characteristics of diabetic peripheral nerve injury, indicating that the axons are more prone to early damage.

The mechanisms of retinal neurodegeneration in diabetic patients are complex, including ocular and systemic factors (7, 20, 21). Hyperglycemia is one of the most important risk factors, which can destroy the micrometabolic environment in the retina and reduce the signals from the insulin receptor required for nerve growth, development, and survival, eventually leading to nerve cell apoptosis. Although the patients in the present study with obvious blood glucose fluctuations did not have microvascular lesions of early clinical significance, poor blood glucose control can lead to retinal nerve damage in early stages of diabetes without visual impairment. Thus, it can be concluded that blood glucose fluctuation is more likely to cause structural nerve damage before causing vision changes in the early stages of diabetes. Structural neurogenic changes might be the earliest detectable signs in the eyes of diabetic patients. Retinal ganglion cells and other retinal neurons are associated with a phenomenon called “metabolic memory,” where early hyperglycemia is still harmful to nerves, irrespective of whether later glycemic control is improved (22). Therefore, controlling the blood glucose level in the early stages of diabetes and reducing blood glucose fluctuations are critical in the maintenance of long-term retinal health in diabetic patients.

In all, diabetic patients with poor blood glucose control and good vision showed a trend of RNFL and GCL+ thinning irrespective of the absence of retinal microangioma detected by FFA. Diabetes is associated with the potential development of retinal nerve damage. OCT examination can be regularly conducted in the early stages of diabetes to detect RNFL and neuronal changes, as well as the different characteristics of neurodegenerative change. Our findings will contribute to the early screening of retinal neurodegenerative changes in T2DM. This is of great significance for the determination of the degree of retinal nerve damage and the protection of retinal nerve in diabetic patients.

## Data Availability Statement

All datasets generated for this study are included in the article.

## Ethics Statement

The studies involving human participants were reviewed and approved by Ethics committee of the Third Affiliated Hospital of Sun Yat-Sen University. The patients/participants provided their written informed consent to participate in this study.

## Author Contributions

XJ designed the protocol. TB did the OCT examinations and the fundus fluorescein angiography (FFA) examination. ZZ and SW did the slit lamp and intraocular pressure examinations. TJ and YZ checked the data of the patients. QL did the Statistical analysis. XZ and ZZ edited the manuscript. All authors contributed to the article and approved the submitted version.

## Conflict of Interest

The authors declare that the research was conducted in the absence of any commercial or financial relationships that could be construed as a potential conflict of interest.
